# Bending and punching characteristics of aluminum sheets using the quasi-continuum method

**DOI:** 10.3762/bjnano.13.108

**Published:** 2022-11-10

**Authors:** Man-Ping Chang, Shang-Jui Lin, Te-Hua Fang

**Affiliations:** 1 Department of Mechanical Engineering, National Kaohsiung University of Science and Technology, Kaohsiung 80778, Taiwan

**Keywords:** nano-punching, quasi-continuum method, single-crystalline aluminum

## Abstract

The nano-punching characteristics of single-crystalline aluminum are investigated using the quasi-continuum (QC) method. Four variables (i.e., crystal orientation, workpiece thickness, clearance between the punch and the substrate, and the taper angle of punch) are used to explore their effect during the nano-punching process. The shear stress distribution is used to express the punching effect on the punch and on both sides of the substrates. Besides, fracture strength, residual flash, and the atomic displacement vector are observed and discussed regarding the behaviors of the nano-punching process under various conditions. Based on the results, the Al workpiece with the X[111]Y[−110] orientation presents less lattice resistance during the punching process. Besides, the thickness of the workpiece has a significant effect on the punching quality. Workpieces with thickness values of 5 and 10 Å are more suitable for punching, due to stable loading and unloading stress–displacement curves and less residual flash on the cutting surfaces of these workpieces. In contrast, the effect of clearance has less impact on the punching behaviors of thinner workpieces. However, for thicker workpieces (i.e., 15 and 20 Å), a larger clearance will likely cause more residual flash. Furthermore, the taper angle of the punch should not be larger than 10°, otherwise, it might damage the workpiece and the substrate.

## Introduction

Nanotechnology has greatly improved the development of high-tech industries such as biomedicine, nanoelectromechanical systems (NEMS), environmental science, and semiconductors [1–10]. The increased requirements for advanced nanostructures simultaneously give rise to extensive researches in precision machining techniques, including nanoimprinting lithography (NIL) [11–12], mechanical nano-cutting [13–14], and nano-punching [15]. These techniques achieve high precision at the nano- and submicron scale surfaces and three-dimensional structures [16–17]. Thus, the removal methods of nanoscale materials have become more important, such as the nano-cutting and nano-punching processes, to produce high-precision workpieces with complex features and smooth surfaces [18–21]. However, comparing with a large number of studies exploring various mechanisms related to nano-cutting, there is less research focused on the issues of the nano-punching process. Therefore, in the present study, we will focus on characteristics and mechanisms that may influence the nano-punching process.

Because of the size effect and atomic adhesion between the punch and the workpiece, the nano-punching process may present different behaviors in comparison to the conventional punching process. Besides, since it is very challenging to experimentally control every parameter in the nanoscale, the atomistic computational simulation methods are often used. The most commonly used simulation method is molecular dynamics (MD), which has been conducted to investigate many nanoscale processes [22–23]. Although the MD simulation can be effectively applied to simulations at the nanoscale, it still has limitations [14]. Therefore, in recent years, the multiscale simulation approach, which combines atomistic and continuum simulations, has received more and more attention [24–32].

The idea of the multiscale simulation is to model a system without precisely calculating every atom. The major goal of this method is to efficiently treat the continuous model while maintaining its accuracy [33–44]. The multiscale simulation we used for investigating the nano-punching processing was the quasi-continuum (QC) method, which has been extensively studied in recent decades [45–50]. Wu et al. used the QC method to simulate the fracture and cracking of metallic bilayer materials and found that those materials have lower mechanical strength at a specific structural orientation [51]. Besides, Tran et al. studied the friction and scratch characteristics of pure aluminum by the QC method. The bump width to the bump pitch (W/P) value, scratch depth, surface roughness, and indenter radius were set as variables in order to explore the friction behaviors of different models [52]. Moreover, the QC method based on the embedded-atom method (EAM) potential was adopted to observe the fatigue crack growth and expansion characteristics of single-crystal metals under cyclic loading processes. The results showed that after compressive or shear processes, the materials were strengthened if the initial crack coalesces again. On the contrary, if there is no coalescence, the cracks will rapidly spread and lead to the fracture of the materials [53]. Overall, these studies have confirmed that the QC method has been successfully applied in the fields of nanoscale simulations.

In the present study, the QC method was performed to simulate nano-punching processes. Since the QC method has the benefits of simulating large-scale models and reducing simulation operation time to enhance the simulation efficiency, it accurately captures the atomistic physics while retaining the efficiency of continuum models. A nickel punch and a single-crystalline Al workpiece were used as the punching materials. Models with different crystal orientations, workpiece thicknesses, clearances, and taper punch angles were established. The results of the stress–displacement curve, shear strain distribution, and fracture strength were provided as well as the interfacial mechanics and punching characteristics of each nano-punching model.

## Model and Methodologies

**Figure 1:**
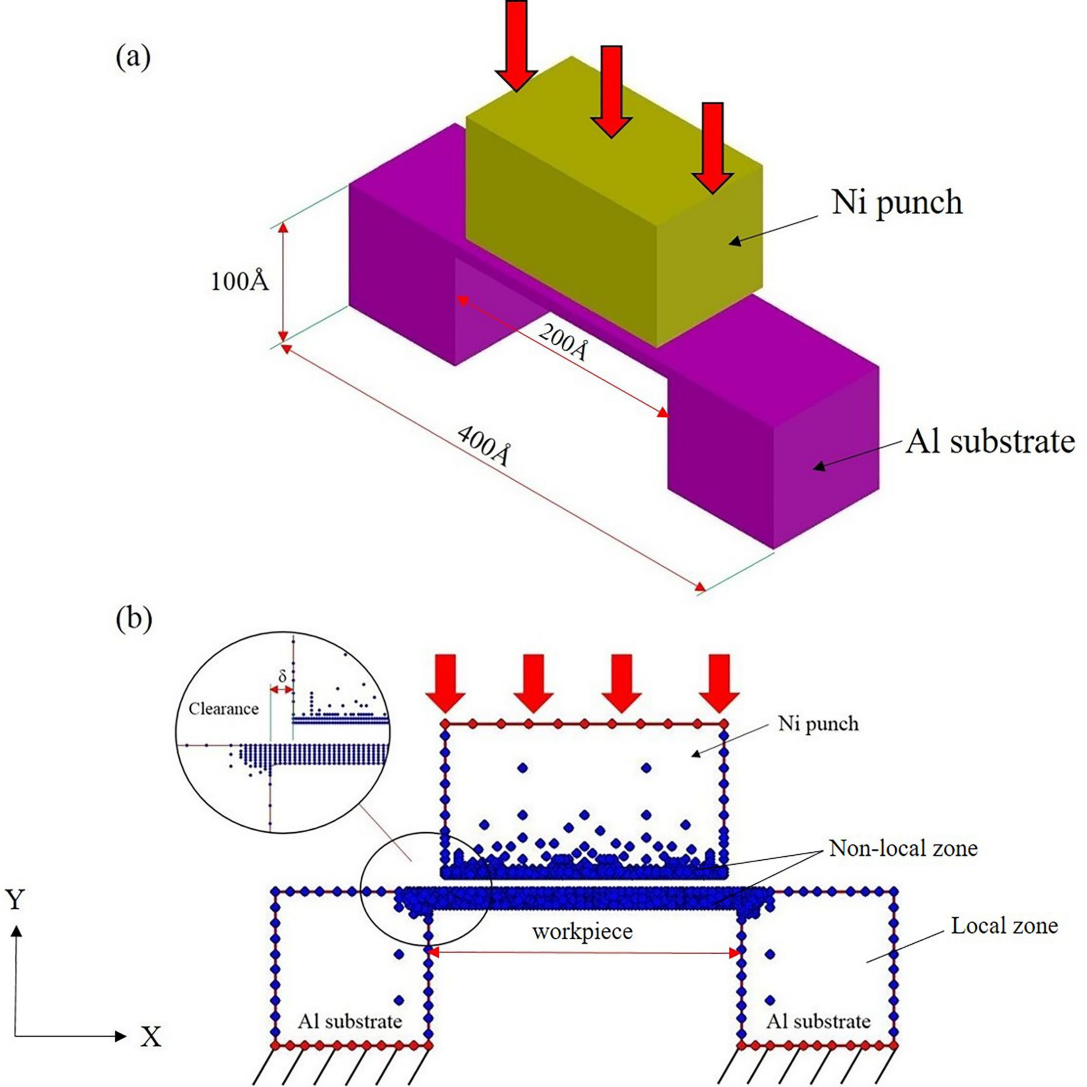
The physical model of the nano-punching system. (a) The punch is made of nickel, and the workpiece is a single crystalline Al. (b) A clearance δ is set between the punch and the substrate during the nano-punching process.

The simulation model of the nano-punching system comprises a punch and a single crystalline Al workpiece, as shown in Figure 1. The punch is assumed as an ideal rigid Ni with crystal orientation of X[−110]-Y[111]. The dimensions of the Al substrates are 100 Å (width) × 100 Å (height), and the width of the Al workpiece is 200 Å, as shown in Figure 1a. A periodic boundary condition (PBC) was applied for the *z*-axis of the simulation model. Besides, there is a clearance between the punch and the substrate during the nano-punching process, as shown in Figure 1b. The initial distance between the punch and the workpiece was set to 10 Å, in order to prevent internal adhesion of atoms in the equilibrium stage which may lead to program error termination. The atoms at the bottom of the Al substrates were the fixed atoms, and the atoms on the top of the Ni punch were the actuation atoms. The nano-punching process was performed by implementing a constant displacement of the Ni punch, which was set to 0.2 Å per step, and the punching displacement was 120 Å. The effects of crystal orientation, workpiece thickness, clearance, and taper angle on the nano-punching process were investigated and analyzed.

The QC method was used to simulate the nano-punching process. In order to improve the computation efficiency, the simulated model was divided into local zone and nonlocal zone, and the atoms that belong to these zones are also called local atoms and nonlocal atoms, respectively. The local atoms represent the continuum region (i.e., the region farther from where the punching takes place) and thus the deformation of this region is uniform [26,50]. The energies of the local atoms are calculated based on the Cauchy–Born rule [14,54], which expresses that the energies of the atoms in the local region are equivalent. Conversely, the nonlocal zone indicates the punching area which suffers the most significant nonuniform deformation. In order to accurately calculate the energy of the nonuniform deformation region, the EAM is used to calculate the interaction of the nonlocal atoms [55]. Moreover, to avoid repeated calculations at the coupled regions, the QC method performs continuous coupled calculations to modify the ghost force of the coupled regions [54].

## Results and Discussion

In this section, the QC method was used to explore the effects of different crystal orientations, workpiece thickness, clearance, and taper angle on the nano-punching process. The friction characteristic of interface and residual flash phenomena were discussed under various conditions. The stress–displacement curve and stress/strain image were used to analyze various mechanical properties.

### Effect of crystal orientation on the nano-punching process

Before discussing the influence of different crystal orientations on the nano-punching process, the effect of crystal orientation on the materials will be firstly considered. The mechanical properties of single-crystal materials can be strongly affected by the crystal orientation [56], such as the elastic stress [57], thermomechanical fatigue behavior [58], and the dislocation effect of deformation caused by an external force [59]. In this work, three crystal orientations O1, O2, and O3 were chosen, which were X[111]Y[−110], X[−110]Y[111], and X[110]Y[001], respectively. For O1, O2, and O3 orientations, the punching directions were parallel, perpendicular, and with a specific angle to the close-packed surface of the single-crystal Al workpiece, respectively [60]. The thickness of the Al workpiece was set to 10 Å and the clearance was 5 Å.

**Figure 2:**
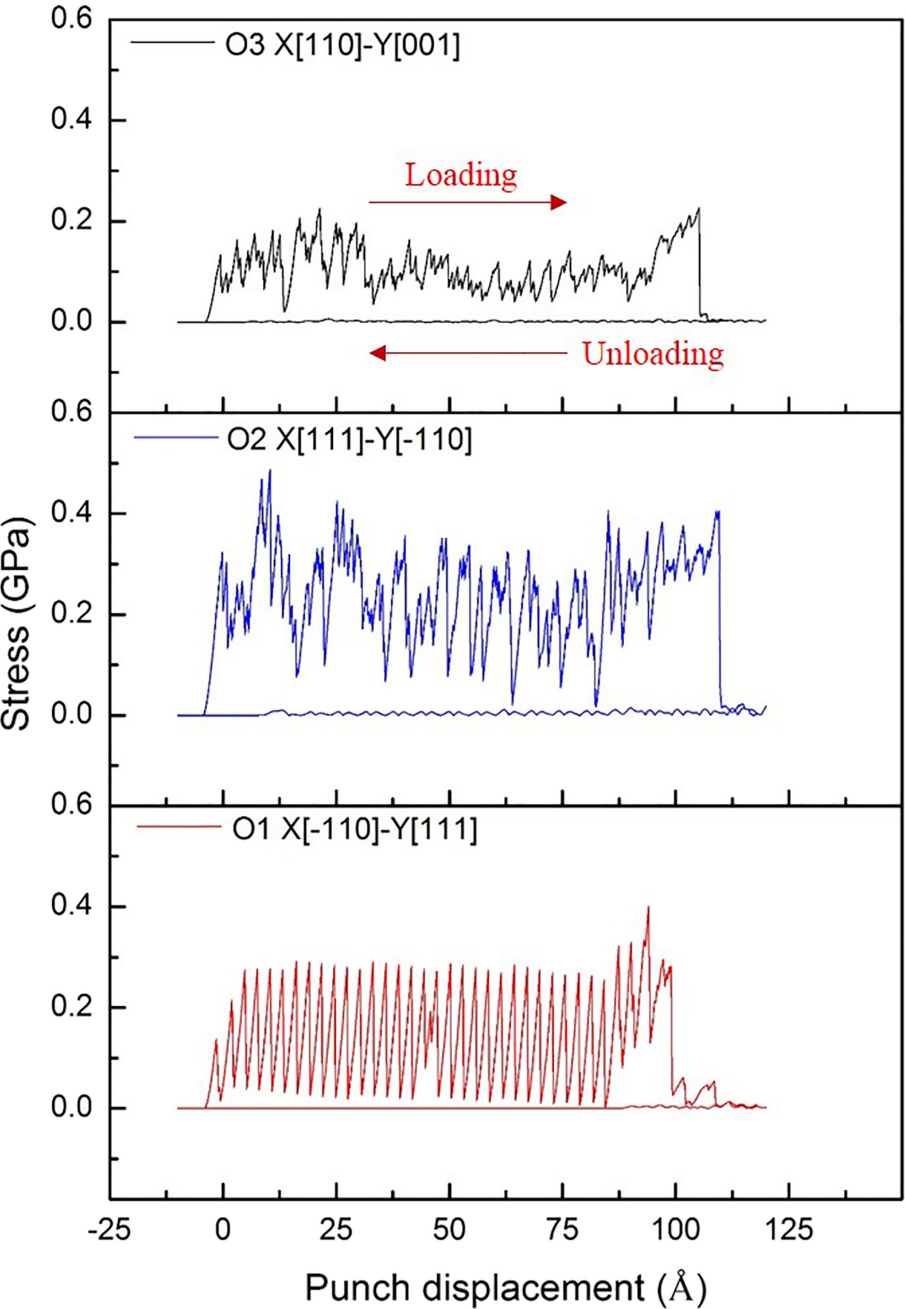
The shear stress–displacement curves of O1, O2, and O3 orientations during the nano-punching process.

**Figure 3:**
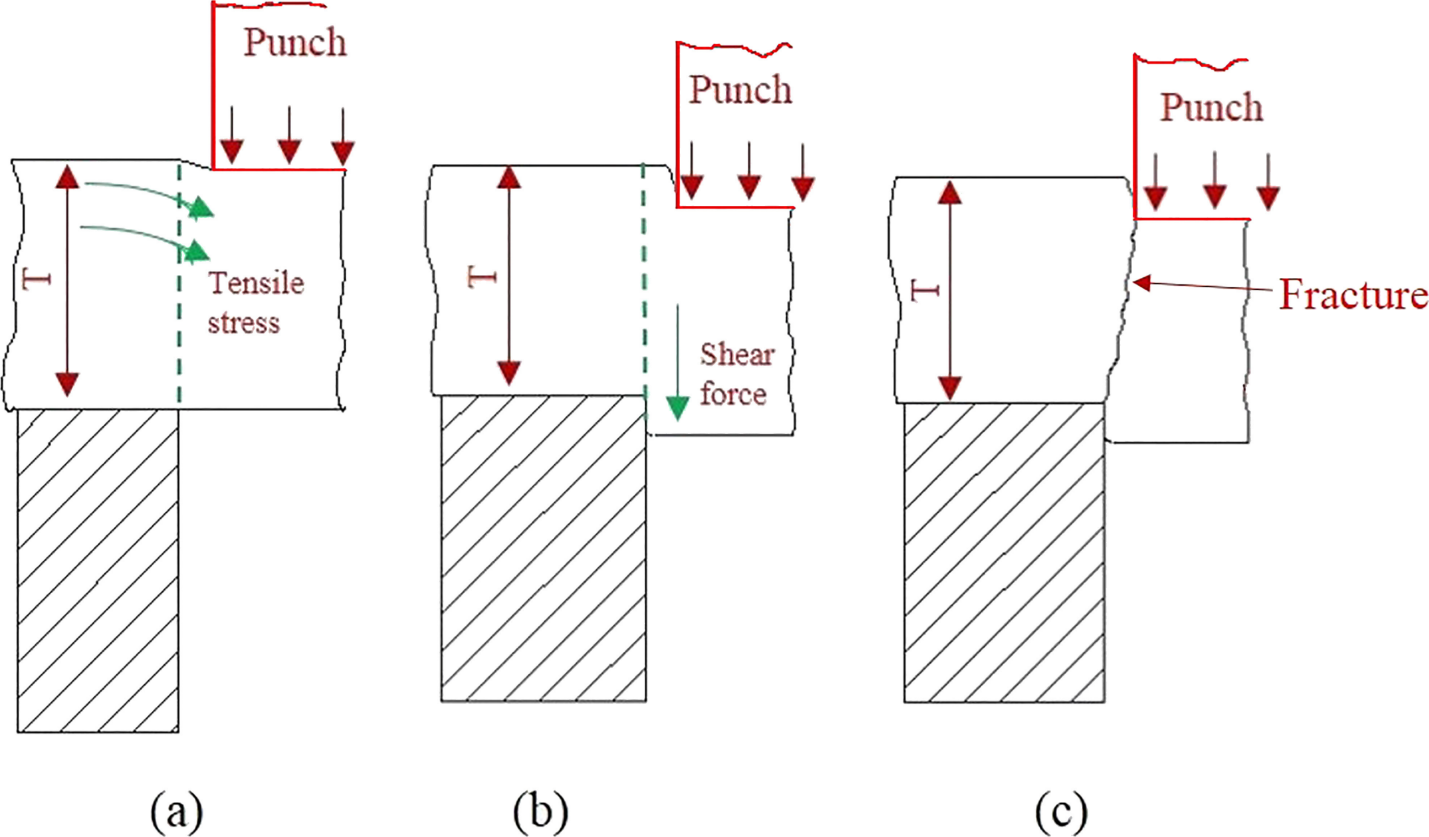
The schematic diagram of the nano-punching process. (a) The elastic deformation stage, (b) the plastic deformation, and (c) the fracture stage.

Figure 2 exhibits the shear stress–displacement curve of O1, O2, and O3 during the nano-punching process. Firstly, it can be observed that a continuous oscillation exists in the curve from the punch contacts with the workpiece to the workpiece fracture. This oscillation expresses the stick–slip phenomenon, which commonly occurs in the atomic-scale friction [14,61–62]. The stick phenomenon is caused by the accumulation of atoms in front of the punch, and the adhesion force increases with an increase of the contact area between the punch and the workpiece. However, when the debris crumple, the slip phenomenon appears [61]. Besides, comparing the three crystal orientation curves, O1 shows a more stable curve during the loading process, while the O2 and O3 curves are less stable. Each of the curves sharply drops at the end, which means that the Al workpiece is broken and separated from the substrate. For a more clear description of the mechanical behavior of the nano-punching process, the schematic diagram of the nano-punching process is shown in Figure 3. Initially, the downward moving punch generates tensile stress between the workpiece and the substrate, causing an elastic deformation inside the workpiece (Figure 3a). When the punch continues to move down until the plastic deformation occurs, there is shear stress between the workpiece and the substrate (Figure 3b). Eventually, the workpiece fractures from the substrate at a critical stress, which is also defined as fracture strength (Figure 3c).

After the workpiece is broken and the loading stage goes toward the end, the punch moves up and returns to the original position. According to the unloading curves shown in Figure 2, it can be found that there is a slight oscillation in the O2 and O3 curves, especially in the O2 curve, which means that the residual flash exists on the interface between the punch and both sides of the substrate. By contrast, there is no oscillation in the unloading curve of O1, which indicates that the unloading process of O1 was less affected by the residual flash. Therefore, the result confirms that there is almost no residual flash on the interface of the O1 orientation. Further, the fracture strength values of O1, O2, and O3 were 0.138, 0.324, and 0.133 GPa, respectively. These values show that during the punching process the lattice resistance of the O2 orientation is significantly higher than that of O1 and O3.

**Figure 4:**
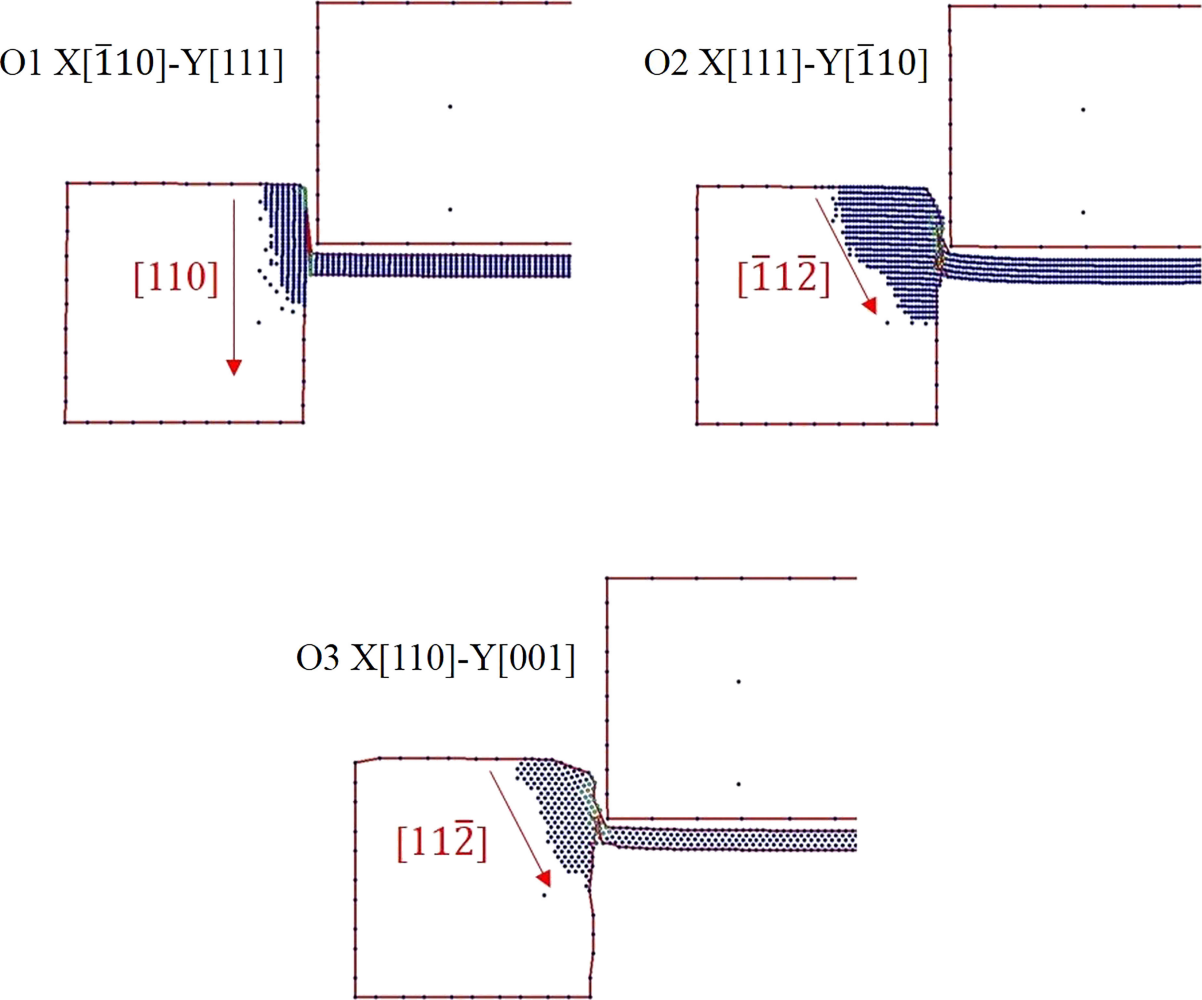
The atomic displacement vectors of O1, O2, and O3 during the punching process.

The different punching behaviors of O1, O2, and O3 can be observed by the atomic vector images, as shown in Figure 4. For the substrate of the O1 orientation, the atoms slip along the [110] direction, which is parallel to the punching direction and thus has a relatively lower resistance during the punching process. Therefore, the stress–displacement curve of O1 presents a relatively stable appearance. By contrast, the atoms slip directions of O2 and O3 are [−11−2] and [11−2], respectively. It can be observed that inclined angles appear on both sides of substrates, and there is an obvious lattice resistance of these substrates during the punching process, results in the unstable stress–displacement curves and more residual flash on the substrates with O2 and O3 crystal orientations.

**Figure 5:**
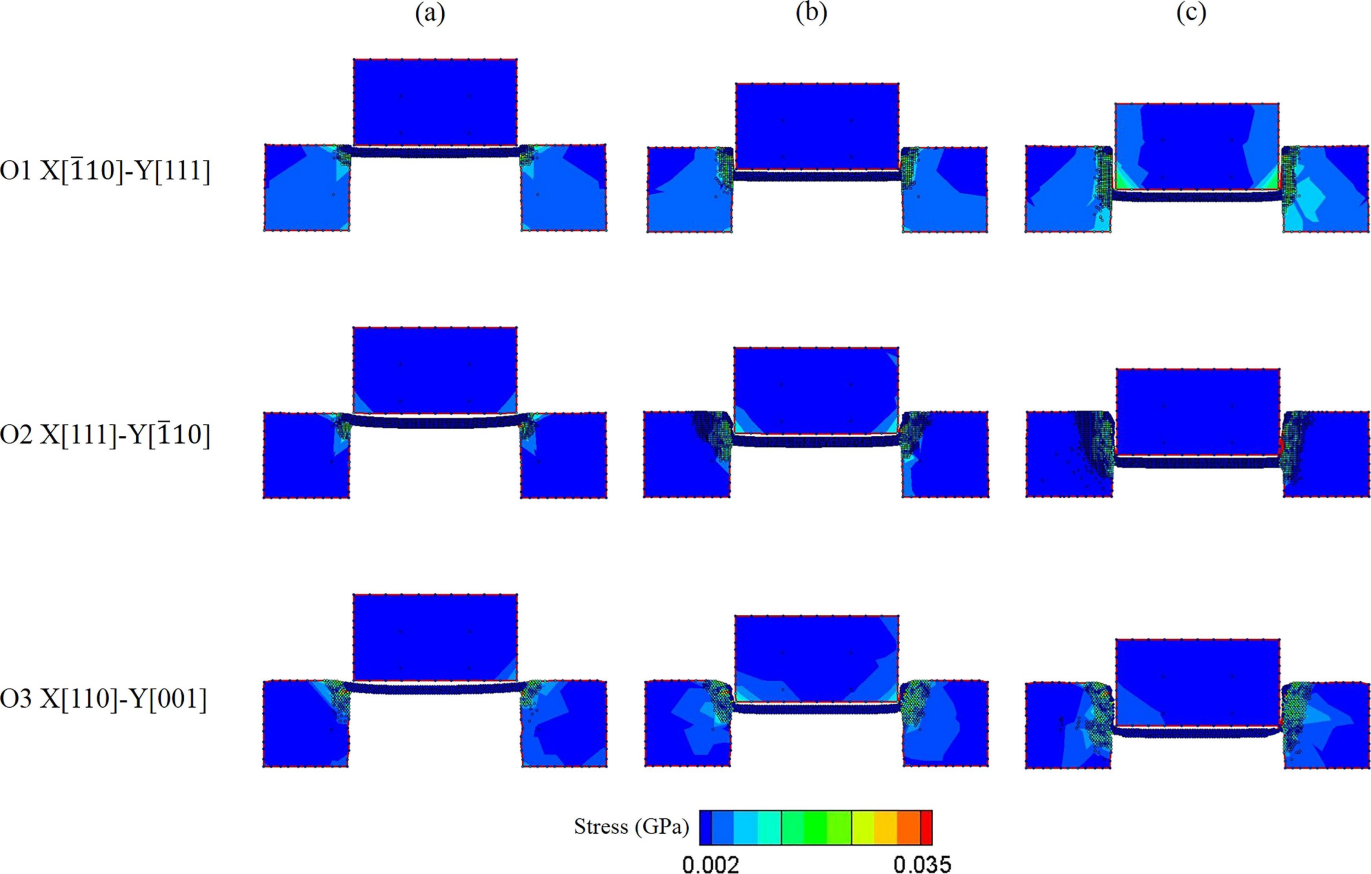
The shear stress distribution diagram of orientation O1, O2, and O3 during the punching process. The displacement of the punch is (a) 0 Å, (b) 25 Å, and (c) 50 Å.

The shear stress value and distribution of the punch and the substrate can be observed in the images. Figure 5 presents the equivalent stress of O1, O2, and O3 crystal orientations during the punching process. It can be found that the stress distribution is obviously different with different crystal orientations. For the O1 orientation, the direction in which the stress is distributed in the punch and both sides of the substrates is pretty much parallel to the punching direction, especially at the displacement *d* = 50 Å. However, the stress distribution of O2 and O3 is at an inclined angle with the punching direction, which is mostly consistent with the atomic displacement vectors mentioned in Figure 4. Besides, it can be more clearly observed from Figure 5 that the cutting surfaces of O1 are relatively smooth. There is no inclined phenomenon or residual flash on the cutting surfaces of the substrates caused by the punching process. By contrast, the substrates of O2 and O3 are inclined due to the force of the punch. The inclination angle of the substrates on both sides of O2 and O3 orientations is approximately 45°, and the stress of the O3 orientation is more widely distributed than that of O2.

**Figure 6:**
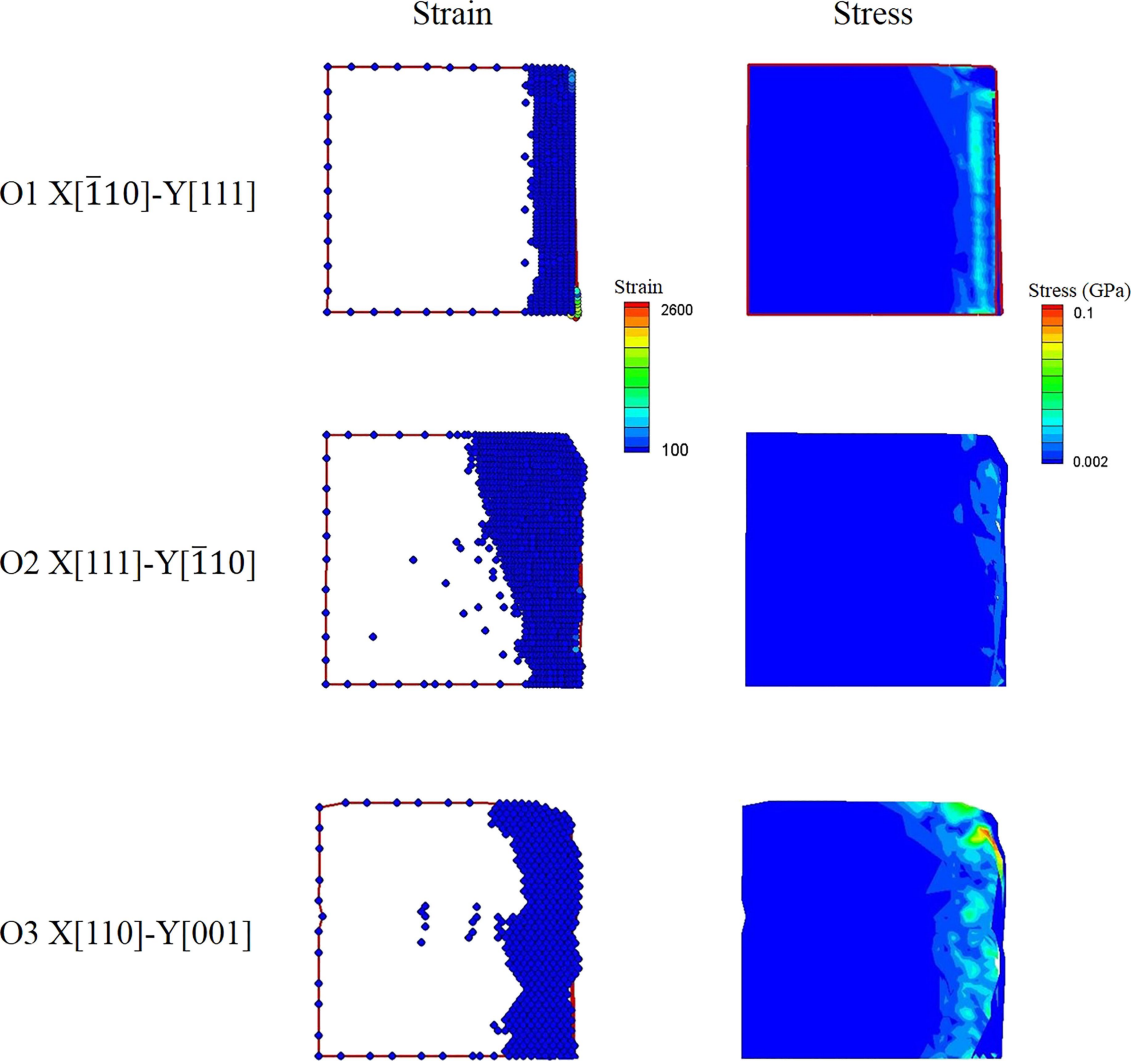
The shear stress and strain distribution during the unloading process of the O1, O2, and O3 orientations.

When the punch displacement was *d* = 120 Å, all of the Al workpieces of the O1, O2, and O3 orientations were broken. Figure 6 shows the shear stress and strain distribution during the unloading process of the O1, O2, and O3 orientations. Because the cutting surface of O1 is smooth, during the unloading stage the punch has very little friction with the cutting surface. Therefore, the unloading stress of O1 is stable at 0 GPa, as shown in Figure 2. In contrast, O2 leaves the largest inclined angle on the substrate, and the strain in the substrate is the highest. However, there is no much residual flash on the cutting surface of O2, so the residual stress of the O2 substrate is low. Compared with O1 and O2, O3 leaves the most residual flash on the cutting surfaces, which leads to the largest friction between the punch and the cutting surface during the unloading process. Therefore, it has the largest residual stress in the O3 substrate.

According to the above results, the difference of crystal orientations of the Al workpiece has shown a great influence on the punching behaviors, which can provide a reference when selecting the punching substrate and workpiece.

### Effect of workpiece thickness on the nano-punching process

After investigating the orientation effect on the nano-punching process, in this section, the effect of various thicknesses of Al workpieces are discussed. The thickness of the Al workpiece was set to *T* = 5, 10, 15, and 20 Å. The clearance between the punch and both sides of the substrate was 5 Å.

**Figure 7:**
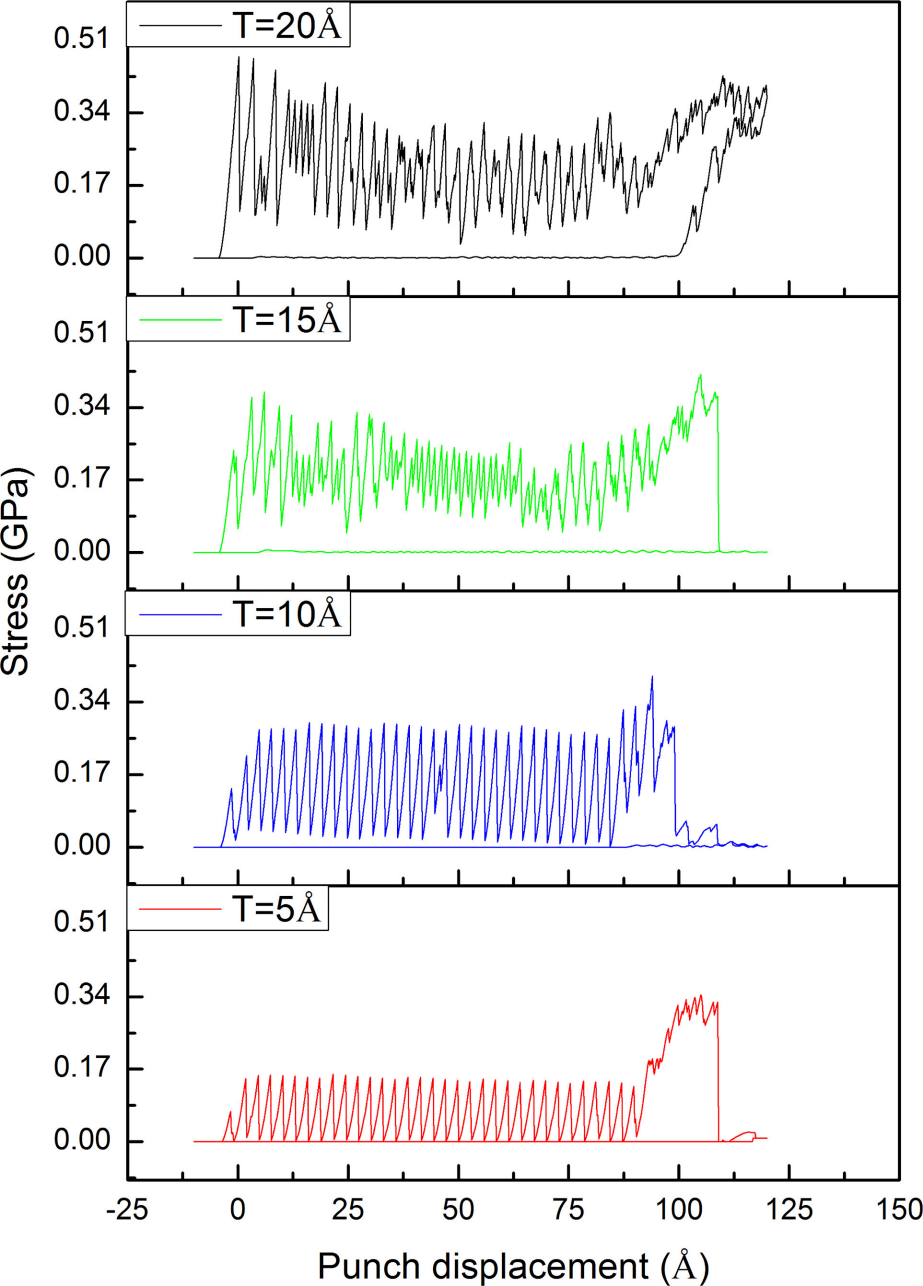
The shear stress–displacement curves of workpieces with various thicknesses during the nano-punching process.

**Figure 8:**
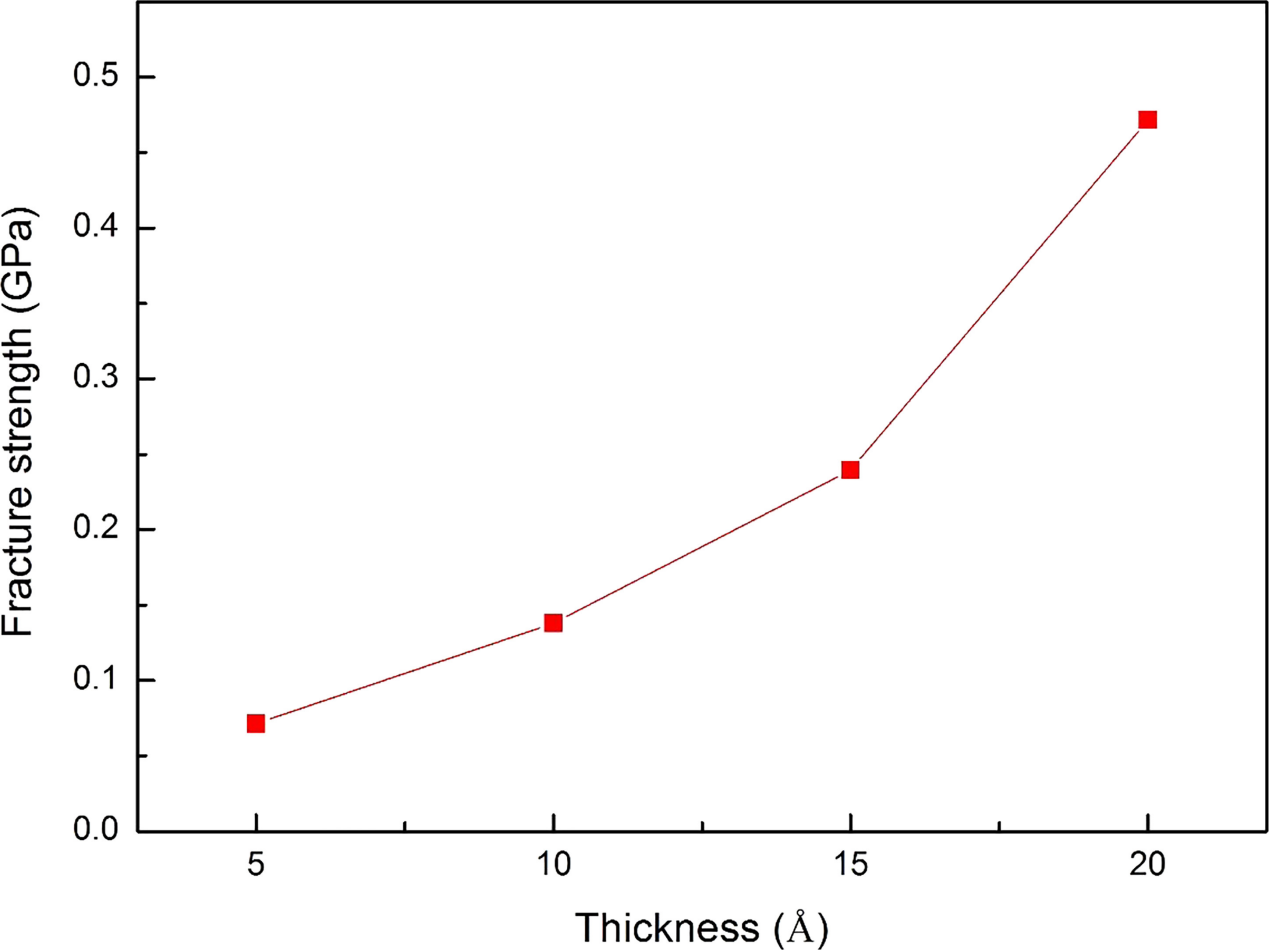
The fracture strength of the 5, 10, 15, and 20 Å workpieces.

Figure 7 exhibits the shear stress–displacement curve of the workpiece with different thicknesses. The workpiece thickness *T* = 5 and 10 Å curves are stable and only show a significant rise when the workpiece is broken. By contrast, the stress curves of *T* = 15 and 20 Å show unstable behavior. Moreover, the 20 Å curve does not show a sharp drop at the end of the punching process like the other curves, which means that the 20 Å workpiece cannot be completely separated from the substrate at the end of the punching process. This result indicates that the 20 Å Al workpiece is too thick for this nano-punching process, which may lead to the failure of the punching process and wearing of the punch. Besides, the influence of the workpiece thickness is also directly displayed on the fracture strength. The fracture strength values of 5, 10, 15, and 20 Å workpieces are 0.072, 0.138, 0.240, and 0.472, respectively, as shown in Figure 8. It indicates that the thicker the workpiece, the greater the punching force must be to make the workpiece fracture from the substrate.

**Figure 9:**
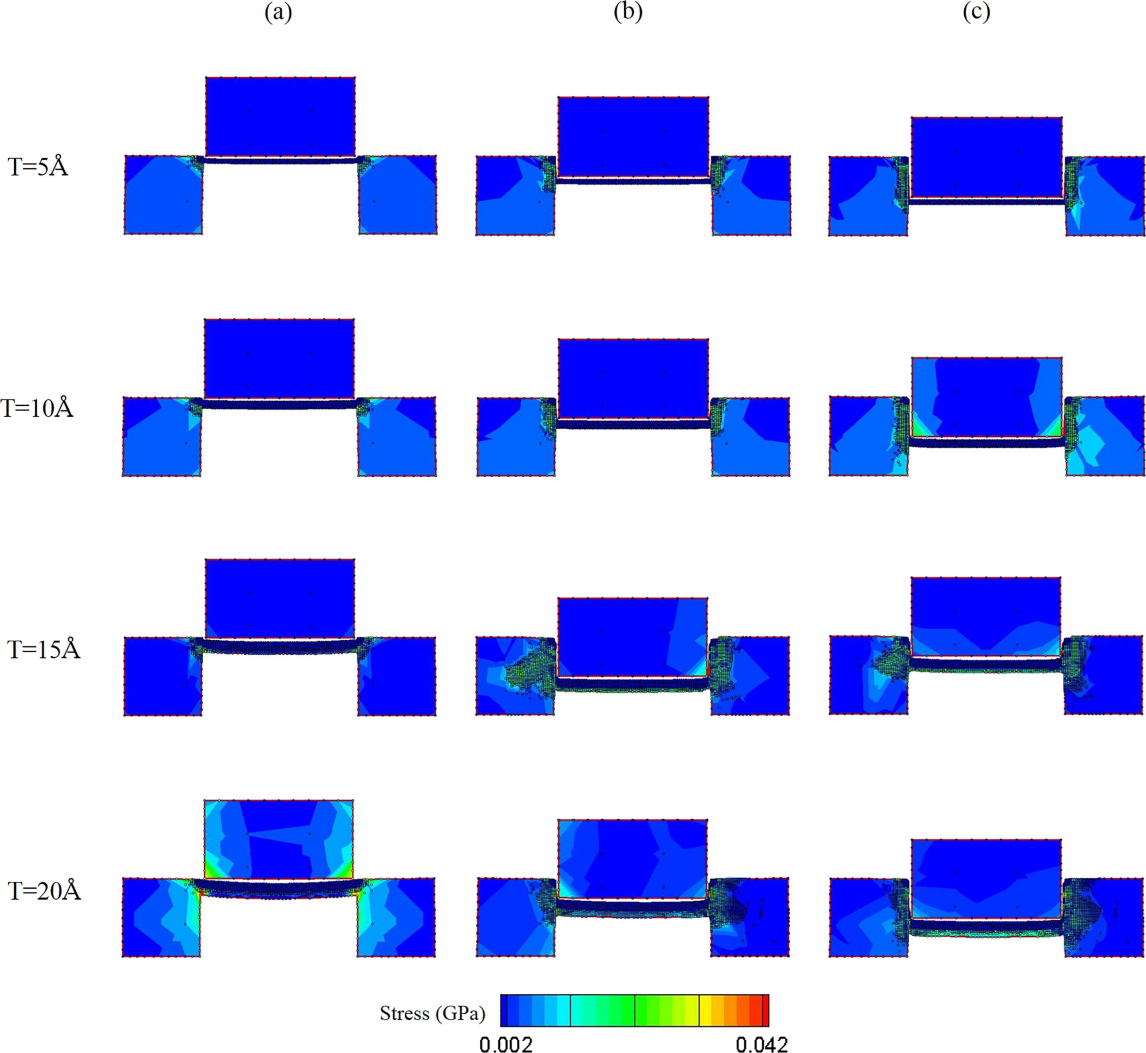
The shear stress distribution diagram of the 5, 10, 15, and 20 Å workpieces during the punching process. The displacement of the punch is (a) 0 Å, (b) 25 Å, and (c) 50 Å.

Figure 9 presents the stress distribution for workpieces with various thicknesses. For the 5 and 10 Å workpieces, during the punching process, the stress uniformly distributes on the interface between the punch and both sides of the substrates. In addition, there is no stress distribution in the punch of the 5 Å workpiece, which means that the workpiece with 5 Å thickness will cause very little force on the punch during the punching process. However, for the 15 and 20 Å workpieces, the stress is distributed in the center of the substrates. Especially for the 20 Å workpiece, when the punch displacement is *d* = 0 Å, there is a relatively great stress distribution in the punch and the substrates. This phenomenon is caused by the greater friction between the thicker workpiece and the punch. Besides, the 20 Å workpiece also suffers great shear stress during the punching process (*d* = 50 Å), because the thicker the workpiece, the greater the friction force between the interface of the workpiece and the substrates, which causes shear stress in the workpiece. Furthermore, as shown in Figure 7, the unloading curves of 15 and 20 Å have slight oscillations, which means that the residual flash exists between the punch and the substrates. Therefore, according to the above results, the 15 and 20 Å workpieces are too thick for this punching process.

### Effect of clearance on the nano-punching process

Having discussed the effect of workpiece thickness, the purpose of this section is to explore the effect of clearances on the nano-punching process. Without a suitable clearance size, the punch life and cutting quality decrease. Therefore, the effect of different clearance sizes on workpieces of various thicknesses is considered. The clearance between the punch and both sides of the substrate was set to δ = 5, 10, 15, and 20 Å. The thickness of the Al workpiece was set to *T* = 5, 10, 15, and 20 Å.

**Figure 10:**
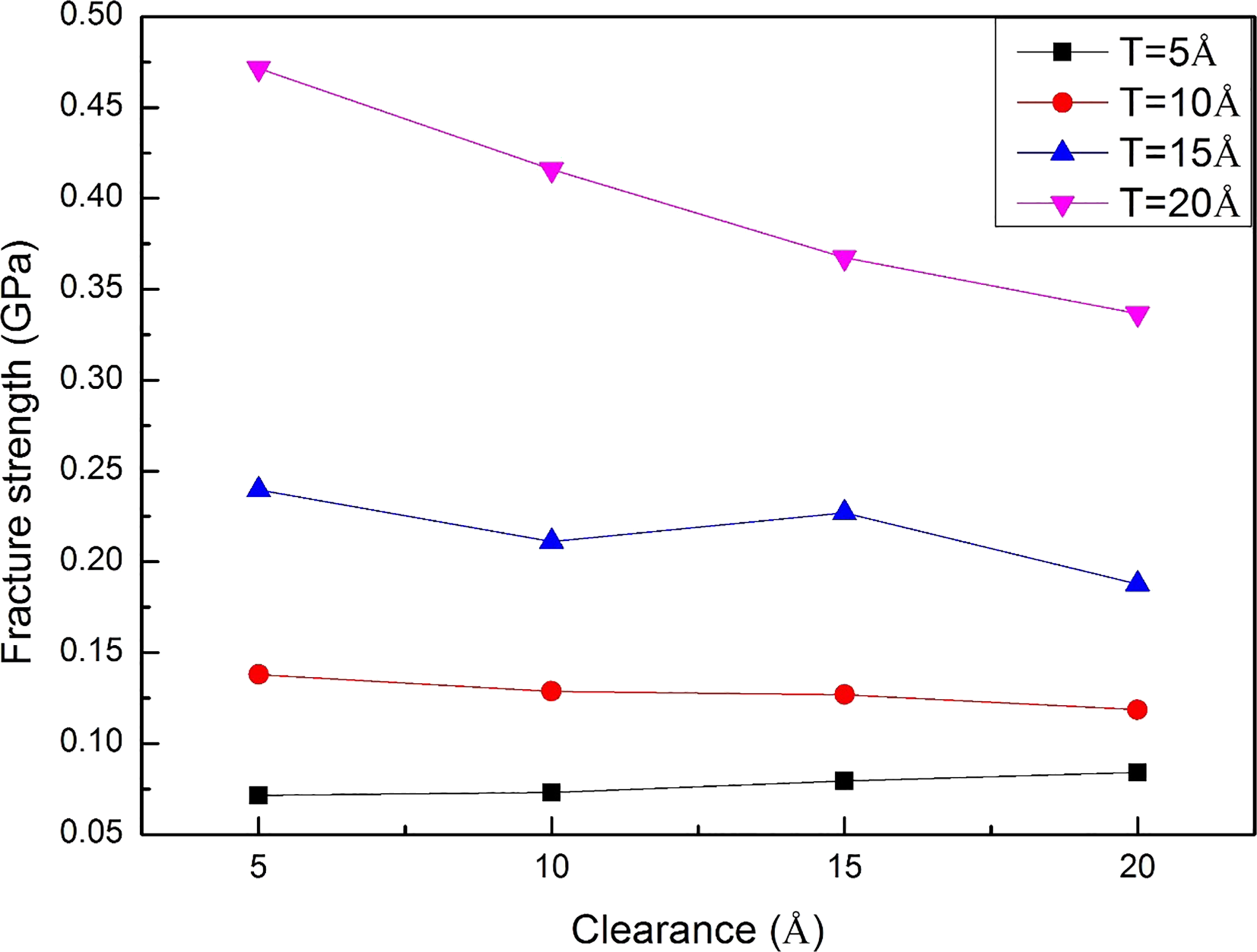
The fracture strength of various workpiece clearances.

**Figure 11:**
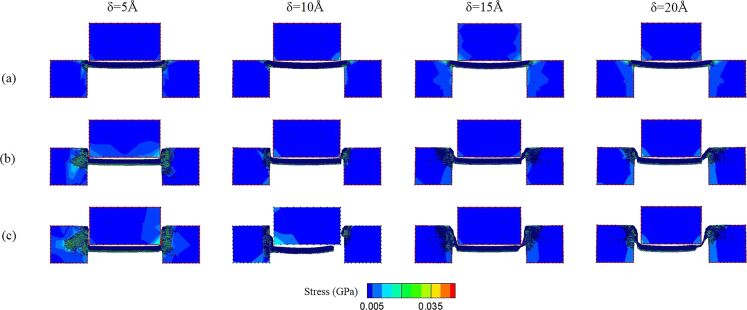
The shear stress distribution diagram of the 15 Å workpiece with 5, 10, 15, and 20 Å clearance values during the punching process. The punch displacement is (a) 0, (b) 25, and (c) 50 Å.

**Figure 12:**
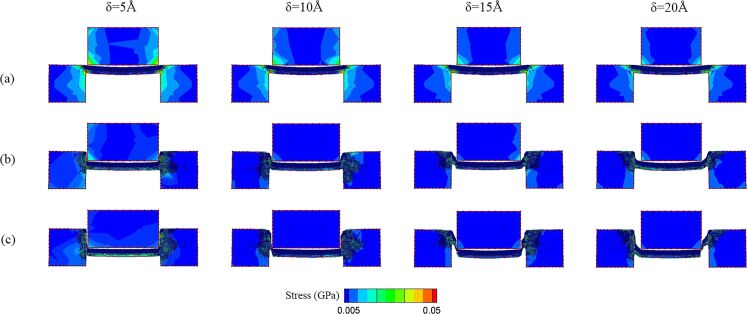
The shear stress distribution diagram of the 20 Å workpiece with 5, 10, 15, and 20 Å clearance values during the punching process. The punch displacement is (a) 0 Å, (b) 25 Å, and (c) 50 Å.

The fracture strength values of workpieces with 5–20 Å clearances are shown in Figure 10. As mentioned above, the fracture strength of thicker workpieces are generally higher than that of the thinner ones. Besides, the fracture strength of 5 and 10 Å workpieces does not show a clear correlation with different clearances. It should be inferred that since these two workpieces are thinner, the size of the clearance does not particularly affect the difficulty of the punching process. In comparison, the fracture strength of thicker workpieces decreases with an increase of clearance, especially for the 20 Å workpiece. In order to further investigate the punching behaviors of the 15 and 20 Å workpieces, the punching process and the stress distribution of the 15 and 20 Å workpieces are observed, as shown in Figure 11 and Figure 12. It can be observed that for the 15 and 20 Å workpieces, as the clearance increases, the value of the stress in the punch and substrates decreases, and the stress distribution is concentrated near the interface where the punching occurs. In addition, under the same punch displacement, the larger the clearance, the larger the area of the workpiece break from the substrate, which explains the smaller fracture strength of these workpieces. These phenomena are more obvious in the 20 Å workpiece than in the 15 Å one. Figure 11c shows the 15 Å workpiece with a 10 Å clearance fracture at a displacement of 50 Å. As it can be seen, the fracture strength of the 10 Å clearance is slightly lower than that of 5 and 15 Å clearance values. However, even the larger clearance values facilitated the punching of thicker workpieces, the inclination angle on the substrate and the residual flash also increased with an increase of clearance, as shown in Figure 11c and Figure 12c.

### Effect of taper angle on the nano-punching process

So far this paper has discussed the influences of different orientations and thicknesses of workpieces, and also considered the clearance between the punch and substrate. This section focuses on the design of the punch. The taper angle of the punch was set to θ = 5°, 10°, 15°, and 20°. The workpiece thickness was 10 Å, and the clearance was 5 Å.

**Figure 13:**
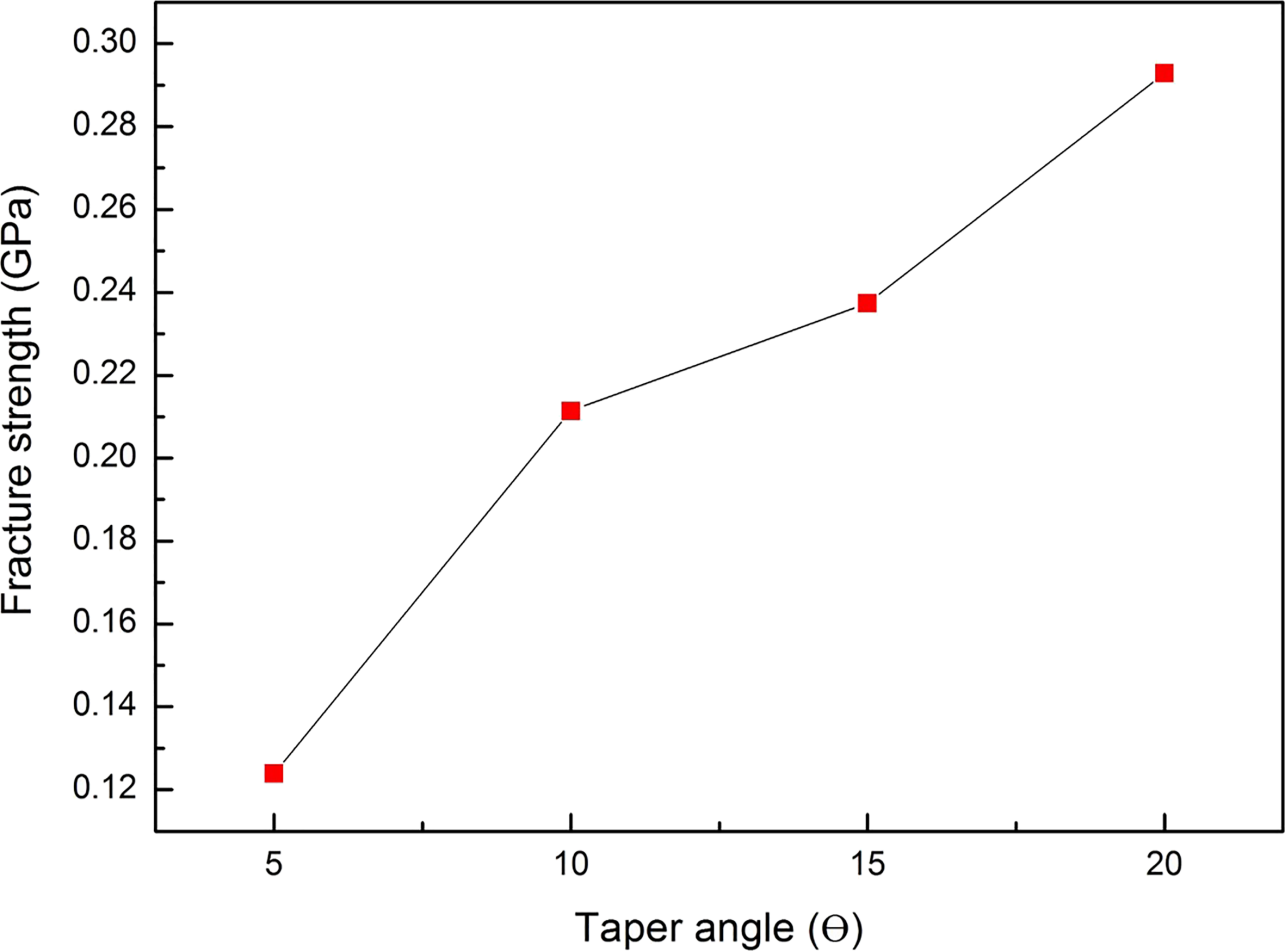
The fracture strength of the punch with various angles.

As shown in Figure 13, the fracture strength values of taper angles θ = 5°, 10°, 15°, and 20° are 0.124, 0.212, 0.237, and 0.293, respectively. This result indicates that the fracture strength is increased with an increase of the taper angle. When the taper angle increases, the contact area between the punch and the workpiece is decreased, and a higher internal energy is stored in the atoms, resulting in a higher fracture strength [63].

**Figure 14:**
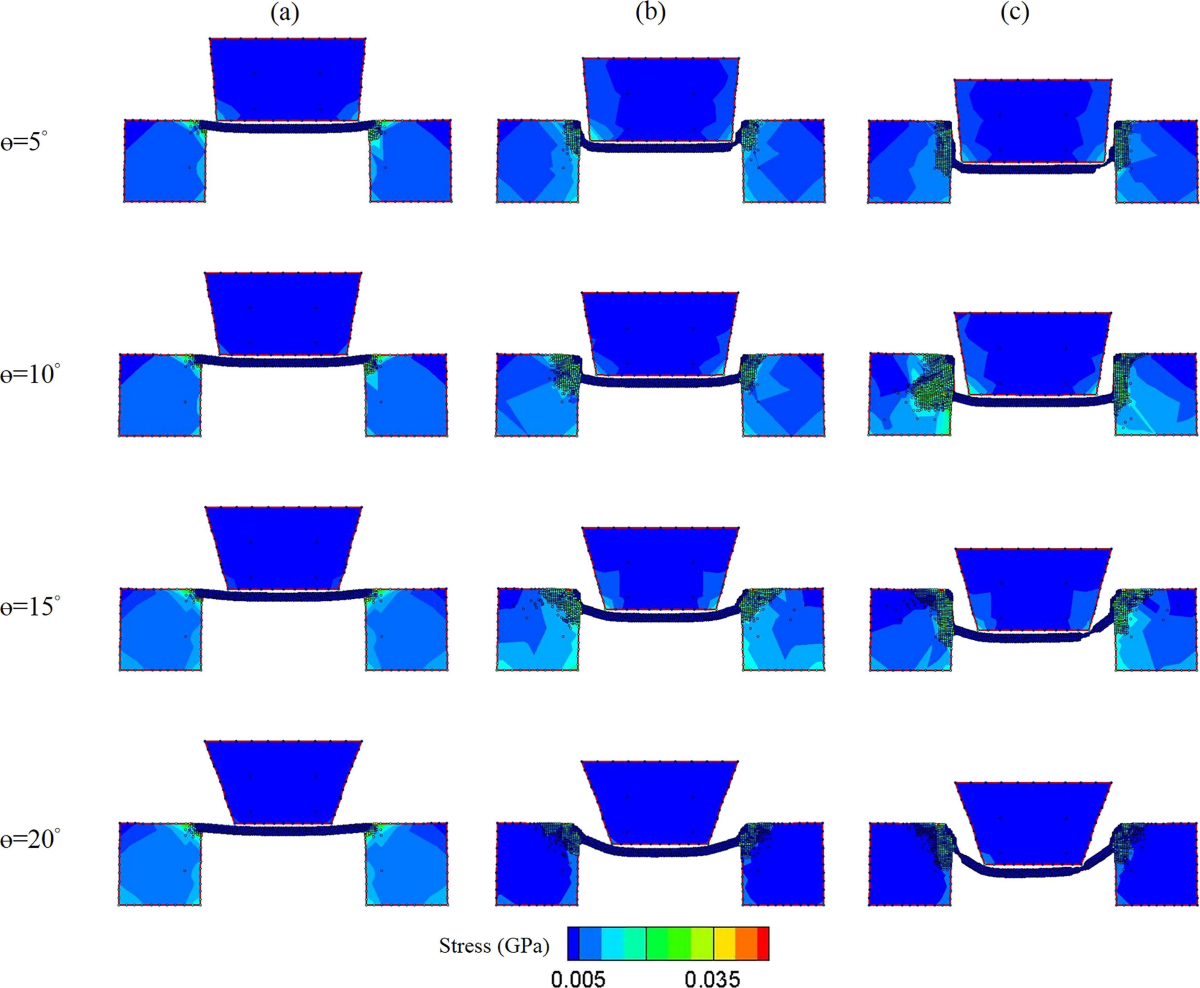
The shear stress distribution of θ = 5°, 10°, 15°, and 20° taper angles during the nano-punching process. The displacement of the punch is (a) 0 Å, (b) 25 Å, and (c) 50 Å.

Figure 14 shows the shear stress distribution of various taper angles during the nano-punching process. It is observed that the punching behaviors changed with the taper angle during the punching process. When the punch displacement was 0 and 25 Å, the stress distribution in the punch and substrate showed little difference with each taper angle. However, when the punch displacement was 50 Å, the stress distribution of the 20° taper angle was obviously smaller than the other ones. Besides, the workpieces were almost broken by the 15° and 20° taper angle punch. Moreover, there were high inclination angles appearing on both sides of the substrates of 15° and 20° taper angles. These results express that punches with 15° and 20° taper angles will likely damage the workpiece and the substrate during the punching process. Even though there is no inclination angle appearing on the substrate at a 5° taper angle punch, there is still some residual flash attached to the cutting surfaces. According to previous research, this phenomenon is due to a too small taper that hinders the material flow during punching [64]. In contrast, the 10° taper angle punch shows smooth cutting surfaces; therefore, this taper angle is more suitable for this punching process.

## Conclusion

In this work, the effects of workpiece orientation, thickness, clearance, and punch taper angle on the nano-punching process of Al workpieces and Ni punches were studied. According to the results shown, several conclusions were obtained. For instance, the crystal orientation of the Al workpiece has a great influence on the punching process. There is no residual flash and inclination angle on the substrate of the O1 X[111]Y[−110] orientation since the punching direction of this orientation was parallel to the close-packed surface of the Al workpiece.

The thickness of the workpiece also plays an important role in the nano-punching process. There is a residual flash between the punch and the substrates of the 15 and 20 Å workpieces. Besides, the 20 Å workpiece cannot be totally separated from the substrate. These results indicate that the 15 and 20 Å workpieces are too thick for the punching process.

Compared to the thickness, the clearance shows little influence on thinner workpieces. However, for thicker workpieces (15 and 20 Å), the inclination angle and the residual flash on the substrate increased with an increase in the clearance.

Different taper angles of punches also affect the punching behaviors. For punches and 10° taper angles, the cutting surfaces were smoother than those for the 5°, 15°, and 20° taper angles. In addition, the workpieces were bent by the 15° and 20° taper angle punches during the punching process.

Based on the presented results, in order to obtain good punching quality, the design of the workpiece and punch should be in a proper range. For this nano-punching process, the 5 or 10 Å thickness Al workpiece with the X[111]Y[−110] orientation, and punch with a taper angle of 10° may be the optimal selection, which can give a reference result for subsequent studies.
